# Supplementary material for: The anthropometric assessment of body composition and nutritional status in children aged 2–15 years: A cross-sectional study from three districts in Bangladesh
Source: PLoS One. 2021 Sep 9;16(9):e0257055. doi: 10.1371/journal.pone.0257055 (PMC8428712; doi:10.1371/journal.pone.0257055)
Supplement: S1 File — (PDF) [file pone.0257055.s001.pdf]

Survey Questionnaire

Dept. of Applied Nutrition and Food Technology
Islamic University, Kushtia-7003, Bangladesh

Date:

Demographic Details

1. Name of the Respondent:
2. Gender:
3. Father's Name:
4. Father's Occupation:
5. Father's Highest Education:
6. Mother's Name:
7. Mother's Occupation:
8. Mother's Education:
9. Address:
10. Best Daytime cellphone No (if applicable):
11. Total Family Income (BDT):
12. Source of Drinking Water:
13. Respondent's Education:
14. Respondent's Occupation:
15. Hand Washing Practice (Before meal and after toilet):

Anthropometric Details

16. Details of Respondent:

Table A

Date of Birth	Age (Y & M)	Height (cm)	Weight (kg)	MUAC (mm)
1st				
2nd				
3rd				
Average				

Table B

	**Skinfold Thickness (mm)**						
	Tricef	Bicef	Subscapular	Suprailliac	Abdominal	Thigh	Calf
1st							
2nd							
3rd							
Average							

____
Name of Interviewer

____
Sign of Interviewer

জরিপ প্রশ্নাবলী

ফলিত পুষ্টি ও খাদ্য প্রযুক্তি বিভাগ
ইসলামিক বিশ্ববিদ্যালয়, কুষ্টিয়া–7003, বাংলাদেশ

তারিখ:

জনসংখ্যার বিবরণ

১। উত্তরদাতার নাম:
২। লিঙ্গ:
৩। পিতার নাম:
৪। বাবার পেশা:
৫। পিতার সর্বোচ্চ শিক্ষা:
৬। মাতার নাম:
৭। মায়ের পেশা:
৮। মায়ের শিক্ষা:
৯। ঠিকানা:
১০। বেস্ট ডেটাইম সেলফোন নং (প্রযোজ্য ক্ষেত্রে):
১১। পরিবারের মোট আয় (বিডিটি):
১২। পানীয় জলের উত্স:
১৩। উত্তরদাতাদের শিক্ষা:
১৪। উত্তরদাতাদের পেশা:
১৫। হাত ধোয়ার অনুশীলন (খাবারের আগে এবং টয়লেট দেওয়ার পরে):

নৃতাত্ত্বিক বিশদ

১৬। বিবরণ: টেবিল এ

	জন্ম তারিখ	বয়স (বছর ও মাস)	উচ্চতা (সে.মি.)	ওজন (কেজি)	বাহুড় ব্যাস (মিমি)
১ম					
২য়					
৩য়					
গড়					

টেবিল বি

	স্কিনফোল্ড থিকনেস (মিমি)						
১ম	ট্রাইসেফ	বাইসেফ	সাবস্ক্যাপুলার	সুপ্রেইলিয়াক	পেটের	উরু	কালফ
২য়							
৩য়							
গড়							

তথ্য গ্রহণকারীর নাম

তথ্য গ্রহণকারীর স্বাক্ষর
