# Supplementary material for: The anthropometric assessment of body composition and nutritional status in children aged 2–15 years: A cross-sectional study from three districts in Bangladesh
Source: PLoS One. 2021 Sep 9;16(9):e0257055. doi: 10.1371/journal.pone.0257055 (PMC8428712; doi:10.1371/journal.pone.0257055)
Supplement: Attachment — Submitted filename: Response to Reviewers.docx [file pone.0257055.s002.docx]

**REBUTTAL LETTER**

16 June 2021

Jose M. Moran
Academic Editor
PLOS ONE

**Ref: Manuscript ID: PONE-D-21-12299**

Manuscript Title: The anthropometric assessment of body composition and nutritional status in children aged 2-15 years: A cross-sectional study from three districts in Bangladesh

Dear Jose M. Moran,

Thank you for your email and for reconsidering our submission. We have addressed the issues raised by the editorial team. A rebuttal letter, which provides our responses, is appended.  We hope that the revised manuscript will prove acceptable for publication.

Kind Regards,

Md Kamruzzaman

(On behalf of co-authors) **POINT-BY-POINT REBUTTAL**

**Response to Editor**

1. Please ensure that your manuscript meets PLOS ONE's style requirements, including those for file naming. The PLOS ONE style templates can be found at

https://journals.plos.org/plosone/s/file?id=wjVg/PLOSOne_formatting_sample_main_body.pdf and

[https://journals.plos.org/plosone/s/file?id=ba62/PLOSOne_formatting_sample_title_authors_affiliations.pdf](about:blank)

*Response: We thank the Associate Editor for this comment. We have checked again the PLOS ONE’S style requirements and ensured the style.*

2. Please provide additional details regarding participant consent. In the ethics statement in the Methods and online submission information, please ensure that you have specified what type you obtained (for instance, written or verbal, and if verbal, how it was documented and witnessed). If your study included minors, state whether you obtained consent from parents or guardians. If the need for consent was waived by the ethics committee, please include this information.

*Response: We thank the editor for this comment. We have revised the section related to participant consent and provided more information (Page 8 and Line 318).*

3. We suggest you thoroughly copyedit your manuscript for language usage, spelling, and grammar. If you do not know anyone who can help you do this, you may wish to consider employing a professional scientific editing service.

*Response: Thanks for this comment. We have copyedited the manuscript language usage, spelling, and grammar. [Edited by Dr Md Rezaul Haque, Professor, Dept. of English, Islamic University, Kushtia, Bangladesh, and* Adjunct Associate Professor at St. John's University, New York, USA*)*

4. Please include additional information regarding the survey or questionnaire used in the study and ensure that you have provided sufficient details that others could replicate the analyses. For instance, if you developed a questionnaire as part of this study and it is not under a copyright more restrictive than CC-BY, please include a copy, in both the original language and English, as Supporting Information.

*Response: We have included the questionnaire in original language (Bengali) and English as supplementary file.*

Furthermore, in your Methods section, please provide a justification for the sample size used in your study, including any relevant power calculations (if applicable).

*Response: Thanks for this comment. We have now included the details of sample size calculations (Page 7, Line 298)).*

5. We note that Figure 1 in your submission contain map images which may be copyrighted. All PLOS content is published under the Creative Commons Attribution License (CC BY 4.0), which means that the manuscript, images, and Supporting Information files will be freely available online, and any third party is permitted to access, download, copy, distribute, and use these materials in any way, even commercially, with proper attribution. For these reasons, we cannot publish previously copyrighted maps or satellite images created using proprietary data, such as Google software (Google Maps, Street View, and Earth). For more information, see our copyright guidelines: http://journals.plos.org/plosone/s/licenses-and-copyright.

We require you to either (1) present written permission from the copyright holder to publish these figures specifically under the CC BY 4.0 license, or (2) remove the figures from your submission:

*Response: Thanks for this comment. We have now replaced the figure* that complies with the CC BY 4.0 license. *(Fig 1) (Page 34, Line 1712).*

**Comments from Reviewer 1**

Reviewer #1: This manuscript presents a very interessant approach about how to evaluate antropometric measures on children and adolescence. The writting is well done , specially on introdution and methods, perphaps some improvements are need on other sections that will be pojnted bellow. In general, the manuscript is eligible to be published, but need some revisions.
Authors states on introduction that they intend to incorporate new antropometric indicators in addition to assessing the nutritional status. It would be very interesting if the could take a more comparative approach of the results found here with the traditional findings in their discussion.
It is of note that they were very cautious on methods since it is it is very well detailed, but please specift why did you decided to do arithmetic average for anatomical point instead of traditional average as you did the other antropometric measures.
The firts paragraph of results (line 237) it is not necessary since the age range of children is very extensive, what can the average add to knowledge? Results b age group and sex is much more plausible. Something similar happens when authors presents the mean wieht comparation between groups by age (line 272) : What is the relevance of information? Children of very different ages will certainly have different weights. Unlike the FMI/ FM comparison that presented great and interesting results that shoul be discussed.
Please, review the discussion thread: do not mention tables and present direct results, use this section to add the new ideas of your study and return to that at the conclusion (the conclusion do not mentioed the use of the new approach on antropometric measures)

FInally, check all commas and points there are serveral

Specific Query and Response

Q1: It would be very interesting if they could take a more comparative approach of the results found here with the traditional findings in their discussion.

*Response: We thank the reviewer for their comments. We have revised the discussion section and provided more information (Page 16).*

Q2: It is of note that they were very cautious on methods since it is it is very well detailed, but please specift why did you decided to do arithmetic average for anatomical point instead of traditional average as you did the other antropometric measures.

*Response: Thanks for this comment. As far as we know, arithmetic mean and traditional mean are same, and we used this arithmetic mean or traditional mean. However, the little (point) difference that you have noticed is due to the use of different statistical package or function that we have used in RStudio. We hope this little difference won.t be a big problem.*

Q 3

The first paragraph of results (line 237) it is not necessary since the age range of children is very extensive, what can the average add to knowledge? Results b age group and sex is much more plausible. Something similar happens when authors presents the mean wieght comparation between groups by age (line 272) : What is the relevance of information? Children of very different ages will certainly have different weights. Unlike the FMI/ FM comparison that presented great and interesting results that shoul be discussed.:

*Response: Thanks for this comment. Average age and weight of children has been deleted from paragraph of result section (Page 11, Line 438).*

Q4: Review the discussion thread: do not mention tables and present direct results, use this section to add the new ideas of your study and return to that at the conclusion (the conclusion do not mentioed the use of the new approach on antropometric measures)

*Response: Thanks for this comment. The discussion section has been revisited and addressed those issues. (Page 16, Page 23).*

Q5 FInally, check all commas and points there are serveral errors on discussion text and on table 1 (mean 2.126 ??)

*Response: Thanks for this comment. All error of commas and points has been checked and corrected. (Page 22, Table 1).*

**Comments from Reviewer 2**

Reviewer #2: This is an interesting study regarding the anthrometry assessment of body composition and nutritional status in Bangladesh children aged 2-15 years. The sample size is relatively small by considering this study was carried out at 3 districts. This kind of study is important in a developing country to understand growing children's nutritional status and body composition as well as their contributing factors.

Major concerns:
Introduction:
It lengthy, with unnecessary literature and without a main focus. Significance of this study was not pointed out in the introduction. First paragraph can be deleted, and please consolidated these lines into two paragraphs only (line76-106; and line108-143). Please add in the previous findings on associations between anthropometric and nutritional status, identify the gaps in current literature, and what is the significance of this study.

Methods:
Please add in the ethics approval number. More importantly, how do you assess children's nutritional status by anthropometric? What are the cut offs to categorize children into several status of malnutrition?
The cut-off in the discussion (line 384-393): should be included in method section
Do you have dietary and food intake data--it would be interesting and would value up this paper by cross-checking the nutritional status that was determined by anthropometric measurement as well as food intake questionnaire

Results is okay with suitable analysis

Discussion:
The authors need to re-write the discussion. The current version is hard for the reader to follow. The 1st paragraph of the discussion is to demonstrate the main findings of this paper. The authors have a tendency to explain the different anthropometric parameters/ methods use to access children's body composition profiles (e.g. line 394-385, line 360-382; line 396-401; line 409-412) and these are not necessary. Instead, authors should focus on the main findings: (1) whether the anthropometric and nutritional status in Bangladesh children is lower/ higher as compared to the other developing countries with similar SES with reasons and recommendation; (2) the binary logistic results that show us some potential factors that contributing in the poor nutritional status in Bangladesh children, and with some scientific arguments in related to the previous findings. In addition, please don't mention (Table XXX) in discussion--this should only mention in Result section.

Minor concern
Please check your English. A lot of typos

Q1: Introduction

It lengthy, with unnecessary literature and without a main focus. Significance of this study was not pointed out in the introduction. First paragraph can be deleted, and please consolidated these lines into two paragraphs only (line76-106; and line108-143).

*Response: The introduction section has been revised accordingly (Page 4).*

Q2: Please add in the previous findings on associations between anthropometric and nutritional status, identify the gaps in current literature, and what is the significance of this study.

*Response: Thanks for this comment. This issue has been addressed (Page 7, Line 278).*

Q3 Methods:
Please add in the ethics approval number. More importantly, how do you assess children's nutritional status by anthropometric? What are the cut offs to categorize children into several status of malnutrition?
The cut-off in the discussion (line 384-393): should be included in method section

*Response: Thanks for this comment. Ethical approval no has been added to the method section (Page 8, Line 327). Details of anthropometric cut-off value has been added in details in anthropometric measurement of the method section (Page 9, Line 358).*

Q4: Do you have dietary and food intake data--it would be interesting and would value up this paper by cross-checking the nutritional status that was determined by anthropometric measurement as well as food intake questionnaire

*Response: Thanks for raising this nice issue. Indeed, it was interesting and valuable to include dietary and food intake data. However, we didn’t collect those kinds of data. This is beyond are study objectives. We will try to include this kind of date for cross validation of nutritional status of children in future study.*

Q5: Results is okay with suitable analysis.

*Response: Thanks for this comment.*

*Q6: Discussion:*

*The authors need to re-write the discussion. The current version is hard for the reader to follow. The 1st paragraph of the discussion is to demonstrate the main findings of this paper. The authors have a tendency to explain the different anthropometric parameters/methods use to access children's body composition profiles (e.g. line 394-385, line 360-382; line 396-401; line 409-412) and these are not necessary.*

*Response: Thanks for this comment. The discussion has been rewritten and checked for error.*

Q7*:* Instead, authors should focus on the main findings: (1) whether the anthropometric and nutritional status in Bangladesh children is lower/ higher as compared to the other developing countries with similar SES with reasons and recommendation.

*Response: Thanks for this comment. The issue has been addressed and discussed in detail in the discussion section. (Page 16, 17, 19, 20, 21)*

Q8*:* (2) the binary logistic results that show us some potential factors that contributing in the poor nutritional status in Bangladesh children, and with some scientific arguments in related to the previous findings.

*Response: Thanks for this comment. The issue has been addressed and discussed in detail in the discussion section. (Page 21, Line 1124)*

Q9: Minor concern, please check your English. A lot of typos *Response: The manuscript has been rechecked for language and typos.*
