# Supplementary material for: The anthropometric assessment of body composition and nutritional status in children aged 2–15 years: A cross-sectional study from three districts in Bangladesh
Source: PLoS One. 2021 Sep 9;16(9):e0257055. doi: 10.1371/journal.pone.0257055 (PMC8428712; doi:10.1371/journal.pone.0257055)
Supplement: Attachment — Submitted filename: Response to Reviewers_V2.docx [file pone.0257055.s003.docx]

**REBUTTAL LETTER**

27 July 2021

Jose M. Moran
Academic Editor
PLOS ONE

**Ref: Manuscript ID: PONE-D-21-12299R1**

Manuscript Title: The anthropometric assessment of body composition and nutritional status in children aged 2-15 years: A cross-sectional study from three districts in Bangladesh

Dear Jose M. Moran,

Thank you for your email and for reconsidering our submission. We have addressed the issues raised by the editorial team. A rebuttal letter, which provides our responses, is appended.  We hope that the revised manuscript will prove acceptable for publication.

Kind Regards,

Md Kamruzzaman

(On behalf of co-authors) **POINT-BY-POINT REBUTTAL**

**Response to Editor**

1. Please review your reference list to ensure that it is complete and correct. If you have cited papers that have been retracted, please include the rationale for doing so in the manuscript text, or remove these references and replace them with relevant current references. Any changes to the reference list should be mentioned in the rebuttal letter that accompanies your revised manuscript. If you need to cite a retracted article, indicate the article’s retracted status in the References list and also include a citation and full reference for the retraction notice.

*Response: We thank the Associate Editor for this comment. The reference has been thoroughly checked again. No retracted article was found among the reference list.*

**Comments from Reviewer 2**

Reviewer #2:

Q1: Introduction

Introduction: Can authors shorten and re-write the 1st two paragraphs in Introduction section? They are lengthy and without a specific focus. 1st paragraph (from line 87-118): should focus on the malnutrition in children at developing countries instead of worldwide, while 2nd paragraph (from line 120-153): please put the focus on the anthropometric assessments in children, as well as the one that you used in the current study, rather than a literature review on all the available methods in human.

*Response: The introduction section has been revised accordingly (Page 15).*

Q2: Discussion:
1st paragraph (line 388-397) I would think line 388-394 are not necessary, suggest removing. Please keep line 394-397 and please summarise the main findings of this study in your 1st paragraph of your discussion section.
*Response: Thanks for this comment. The discussion section has been revised and shorten accordingly Line 388-397 has been removed and main findings of this study has been summarized. (Page 4).*

Q3: Line 399-410, can you summarise the main message from these lines. This is too lengthy

*Response: The section has been revised and summarized accordingly (Page 16, Line 460).*

Q4: Line 413-415: Please check the sentence. Wrong placement for punctuation marks and capital letters.

*Response: The section has been revised accordingly (Page 16, Line 464).*
